# Analysis of physical activity and prescription opioid use among US adults: a cross-sectional study

**DOI:** 10.1186/s12889-024-18220-7

**Published:** 2024-03-05

**Authors:** Junpeng Wu, Panpan Yang, Xiaodan Wu, Xiaoxuan Yu, Fanfang Zeng, Haitang Wang

**Affiliations:** 1 Department of Anesthesiology, Nanfang Hospital, Southern Medical University; The Key Laboratory of Precision Anesthesia & Perioperative Organ Protection, Guangzhou North Road, Guangzhou, 510515 Guangdong China; 2 Department of Anesthesiology, Guangzhou Women and Children’s Medical Center, Guangdong, China; 3 Department of Anesthesiology, Shenzhen Maternity and Child Healthcare Hospital, Southern Medical University, Guangdong, China

**Keywords:** Physical activity, Opioids, Analgesics, NHANES

## Abstract

**Background:**

Opioid crisis has become a global concern, but whether physical activity (PA) can effectively reduce prescription opioid use remains unclear. The study aimed to examine the relationship of different domains of PA (e.g., occupation-related PA [OPA], transportation-related PA [TPA], leisure-time PA [LTPA]) with prescription opioid use and duration of prescription opioid use.

**Methods:**

This cross-sectional study was conducted on 27,943 participants aged ≥ 18 years from National Health and Nutrition Examination Survey (NHANES, 2007– March 2020). We examined the relationship of different domains of PA with prescription opioid use and duration of prescription opioid use using multivariable logistic regression. Stratified analysis and a series of sensitivity analysis were used to elevate robustness. All analyses were conducted using appropriate sampling weights.

**Results:**

Of the 27,943 participants, the mean age was 45.10 years, with 14,018 [weighted, 50.0%] females and 11,045 [weighted, 66.0%] non-Hispanic White. After multivariable adjustment, inverse associations between PA and prescription opioid use were observed for sufficient (≥ 150 min/week) total PA (OR,0.68 95%CI [0.56–0.81]), TPA (OR,0.73 95%CI [0.58–0.92]), and LTPA (OR,0.60 95%CI [0.48–0.75]) compared with insufficient PA(< 150 min/week), but not for sufficient OPA (OR,0.93 95%CI [0.79–1.10]). In addition, the associations were dose-responsive, participants had 22–40%, 27–36%, and 26–47% lower odds of using prescription opioids depending on the duration of total PA, TPA, and LTPA, respectively. Nevertheless, the impact of PA on prescription opioid use varied by duration of opioid use. Sufficient total PA was associated with elevated odds of short-term use of prescription opioids (< 90 days). Comparatively, sufficient total PA, TPA, and LTPA had different beneficial effects on reducing long-term use of prescription opioids (≥ 90 days) depending on the strength of opioids.

**Conclusions:**

This study demonstrated sufficient total PA, TPA, and LTPA were inversely associated with prescription opioid use and varied depending on the duration and strength of prescription opioid use. These findings highlight PA can provide policy guidance to address opioid crisis.

**Supplementary Information:**

The online version contains supplementary material available at 10.1186/s12889-024-18220-7.

## Introduction

Prescription opioids are generally considered the most commonly used and effective analgesics for controlling moderate to severe pain and cancer pain [1]. While prescription opioids provide significant pain relief, their euphoric effects increase the risk of misuse, abuse, addiction, and death [2]. The United States, with only 5% of the world's population, consumed 80% of the global opioid supply [3]. Despite a recent decline in the use of prescription opioids in the United States, opioid-related deaths had increased slightly, with 16,706 deaths due to prescription opioid overdose in 2021 [4]. Long-term use of prescription opioids can also lead to various adverse outcomes, including analgesic tolerance [5], immune suppression [6], gastrointestinal dysfunction [7], and cancer risk [8]. Previous studies have indicated that supply-side factors, such as pharmaceutical company marketing and excessive prescribing by doctors [9–11], play a crucial role in the opioid crisis. With the global prevalence of opioids, the opioid crisis has become a global concern [12].

Numerous researches have shown that physical activity (PA) is associated with better overall health, including weight management [13], alleviation of anxiety or depression [14], and pain relief [15]. Factors such as obesity, mental health disorders, and the severity of pain significantly contribute to the desire for opioid treatment [16–18]. Unfortunately, as of 2016, only 26% of men, 19% of women, and 20% of adolescents in the United States met the recommended PA [19]. PA is complex, consisting of occupation-related PA (OPA), transportation-related PA (TPA), and leisure-time PA (LTPA). It is noteworthy that the impact of PA on health varies across different PA domains. LTPA is considered to play a positive effect on the prevention of most chronic non-communicable diseases (NCDs) [20], whereas high OPA increases the incidence of several NCDs [21, 22]. Furthermore, the NHANES study indicates a higher incidence of LTPA absence among adults with chronic pain, and researchers believe that OPA is often linked to a higher risk of musculoskeletal pain [23–25].

Studies have reported a notable lack of enthusiasm towards PA among individuals undergoing methadone maintenance treatment (MMT) [26, 27]. Only 38% of individuals in MMT achieved the recommended weekly amount of PA [28]. Recent research find that engaging in exercise was more likely to facilitate the discontinuation of opioids within one year of commencing opioid use for pain [29]. Nevertheless, some researchers have suggested that higher occasion-level PA was associated with an increased likelihood of opioid use in patients with chronic low back pain [30]. To date, the association of PA with the risk of prescription opioid use remains unclear, and whether the effect varies by PA domains.

To address this research gap, we used National Health and Nutrition Examination Survey (NHANES) data from 2007 to March 2020 to investigate the relationship between different domains of PA and prescription opioid use. Additionally, we also investigated the association of PA with duration of prescription opioid use. We hypothesized that OPA associates with increased risk of prescription opioid use while the other PA domains associate with reduced risk, and the relationship varied with the duration and strength of prescription opioid use.

## Methods

### Study population

NHANES is a nationally representative cross-sectional survey that utilizes a stratified multistage sampling design to assess the health and nutritional status of the non-institutionalized U.S. population [31]. Participants complete an examination consisting of physiological measurements as well as laboratory (including blood and urine) tests. The research protocol has been approved by the Institutional Review Board of the National Center of Health Statistics (NCHS), and written informed consent has been obtained from all participants. Since the de-identified data analyzed during the current study were publicly available from NHANES, the study did not require any review board approval.

**Fig. 1:**
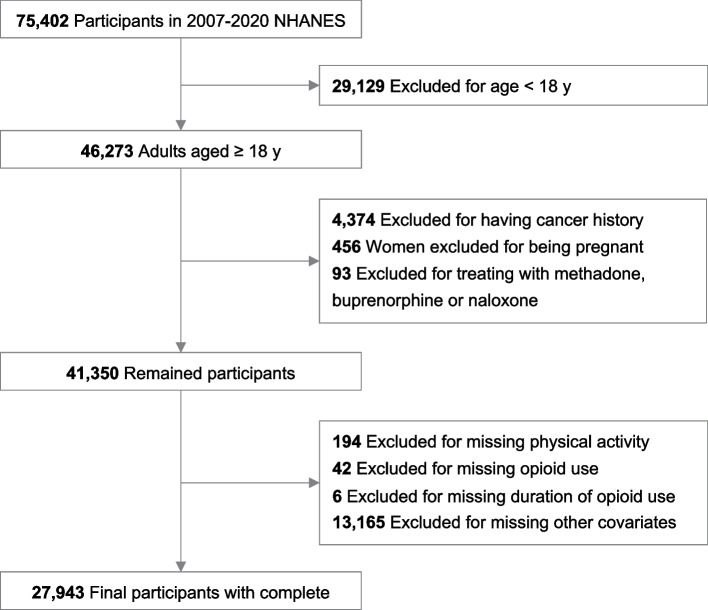
Flowchart of the study participants in NHANES, 2007–2020

This study included participants aged ≥ 18 years from 2007 to March 2020 (*n* = 46,273), after excluding those with a history of cancer (*n* = 4,374), being pregnant (*n* = 456), receiving medications for opioid dependence or withdrawal (including buprenorphine; naloxone, buprenorphine; methadone, *n* = 93), and missing variables (*n* = 13,407), 27,943 participants were included in final. The specific flow was detailed in Fig. 1.

### Assessment of PA

PA was assessed based on the Global Physical Activity Questionnaire (GPAQ), which was the sum of three domains of PA: OPA, TPA, and LTPA. OPA included all work-related PA, continuous walking and cycling that fell under TPA, while all sports, fitness, and recreational activities belonged to LTPA. Participants were asked to report the frequency (days per week), duration (minutes per time), and intensity (vigorous or moderate) of OPA and LTPA, and the frequency and duration of TPA during one typical week. For OPA or LTPA, the minutes of vigorous PA were equivalent to twice that of moderate PA. The amount of each domain of PA was expressed as minutes of moderate PA per week finally [32]. Adhered to the 2018 PA guidelines, we categorized participants into insufficient PA (< 150 min/week) and sufficient PA (≥ 150 min/week) for the main analysis [19]. To explore the dose–response effect of PA with prescription opioid use and to assess the additional benefit of PA above or below PA guidelines, the duration of PA was divided into four groups (0, 1–149, 150–299, and ≥ 300 min/week) [33].

### Prescription opioid use

During the home interviews, participants were asked if they had taken any prescription medications in the past month. Participants who answered "yes" provided the names of the medications through medication containers (82.9%), verbal reports (15.2%), or pharmacy receipts (1.9%), and reported the duration of use for each medication. The investigators classified the medications using the Multum Lexicon therapeutic classification system. Based on previous researches [34, 35], the use of narcotic analgesics or narcotic analgesic combinations was considered as prescription opioid use (excluding methadone, naloxone, and buprenorphine), otherwise it is considered as the non-use of prescription opioids. Long-term opioid treatment can increase the risk of adverse outcomes like opioid dependence,we further divided the duration of prescription opioid use into long-term use (≥ 90 days) and short-term use (< 90 days) [33]. Additionally, prescription opioids were categorized into three strength levels (weaker/equivalent/stronger than morphine) based on morphine equivalency criteria (Table S1).

### Covariates

We identified the following covariates building upon the literature and directed acyclic graph (Figure S1), including age, sex, race/ethnicity (non-Hispanic White, non-Hispanic Black, Mexican American, and other races based on self-report), education (less than high school, high school, and more than high school), poverty-income ratio (PIR, ≤ 130%,130%-300%, and > 300%), cotinine, insurance, alcohol user was categorized as never (< 12 drinks in a lifetime), former (≥ 12 drinks in a year or ≥ 12 drinks in a lifetime but none in the past year), current mild (women: < 2 drinks/d, men: < 3 drinks/d), current moderate (women: 2 to < 3 drinks/d, men: 3 to < 4 drinks/d, or 2–4 binges/month), and current heavy (women: ≥ 3 drinks/d, men: ≥ 4 drinks/d, or ≥ 5 binges/month) [36, 37], and survey cycle.

### Statistical analysis

All analyses were conducted using appropriate sampling weights. Continuous variables were presented as means (standard errors), while categorical variables were presented as frequencies (weighted percentages). Multivariable logistic regression models were used to assess the relationship between PA and prescription opioid use. The insufficient PA was the reference group in this model. No adjustments were made for the model 1; the model 2 was adjusted for age, sex, race/ethnicity; the model 3 was further adjusted for education, PIR, insurance, cotinine, alcohol user, and survey cycle. Additionally, we also performed multivariable logistic regression to assess the relationship between duration of PA and prescription opioid use. The 0 min/week was the reference group in this model. In secondary analyses, we explored the associations of PA with duration of prescription opioid use (no opioid use vs short-term opioid use or long-term opioid use), where long-term opioid use was further divided into using weaker than morphine and equal to morphine or stronger. Short-term use of prescription opioids were not further classified different strength of opioids as only 49 participants had short-term use of prescription opioid weaker than morphine. Furthermore, we conducted stratified analyses by examining age, sex, and race/ethnicity. We also performed several sensitivity analyses. (1) Excluded participants taking benzodiazepines and selective serotonin reuptake inhibitors (SSRIs) antidepressants due to their impact on exercise performance. (2) Defined prescription opioid use ≥ 30 days as long-term use for reanalysis. (3) Estimated the E-values of the association between PA and prescription opioid use, considering potential unmeasured confounders. (4) Multiple imputation method was used to impute the missing values of covariates.

All statistical analyses were performed using R Studio version 4.2.3 (Boston, Massachusetts, USA). It was considered statistically significant if the 95% confidence interval (CI) did not include an odds ratio (OR) value of 1 (two-tailed *p* < 0.05).

## Results

### Characteristics of participants

Of the 27,943 participants, the mean age was 45.10 years, with 14,018 [weighted, 50.0%] females and 11,045 [weighted, 66.0%] non-Hispanic White. Prescription opioid use accounted for 1,606 [weighted, 5.5%], with 244 [weighted, 0.9%] participants reported short-term opioid use, 471 [weighted, 1.4%] participants reported long-term use of prescription opioid weaker than morphine, and 891 [weighted, 3.2%] participants used prescription opioid equivalent to morphine or stronger for long-term. 17,600 [weighted 67.8%], 10,234 [weighted 40.4%], 3,930 [weighted 12.7%] and 9,835 [weighted 39.9%] achieved the recommendations (PA ≥ 150 min/week) for total PA, OPA, TPA, and LTPA, respectively (Table 1).

**Table 1:** Baseline characteristics of participants in NHANES, 2007 to 2020

Characteristics	Participants, No. (Weighted %) (*N* = 27,943)
Age, mean (SE), y	45.10 (0.2)
Sex	
Female	14,018 (50.0)
Male	13,925 (50.0)
Race/ethnicity	
Non-Hispanic White	11,045 (66.0)
Non-Hispanic Black	6,073 (10.8)
Hispanic	2,900 (6.0)
Mexican American	4,272 (9.0)
Other races	3,653 (8.2)
Education	
Less than high school	2,397 (4.3)
High school	10,366 (33.8)
More than high school	15,180 (61.9)
Poverty-income ratio	
≤ 130%	8,951 (21.4)
130%-300%	8,800 (28.2)
> 300%	10,192 (50.3)
Any insurance	
No	6,034 (17.2)
Yes	21,909 (82.8)
Alcohol user	
Never	3,989 (10.5)
Former	3,415 (9.7)
Mild	9,644 (37.3)
Moderate	4,715 (19.0)
Heavy	6,180 (23.5)
Prescription opioid use	
No	26,337 (94.5)
Yes	1,606 (5.5)
Duration and strength of prescription opioid use^a^	
No opioid use	26,337 (94.5)
Short-term opioid use	244 (0.9)
Long-term use of prescription opioid weaker than morphine	471 (1.4)
long-term use of prescription opioid equal to morphine or stronger	891 (3.2)
Total PA, mean (SE), min/week	1053.26 (17.6)
OPA, mean (SE), min/week	741.37 (17.1)
TPA, mean (SE), min/week	73.39 (2.9)
LTPA, mean (SE), min/week	238.50 (5.2)
Total PA: sufficient^b^	17,600 (67.8)
OPA: sufficient^b^	10,234 (40.4)
TPA: sufficient^b^	3,930 (12.7)
LTPA: sufficient^b^	9,835 (39.9)
Cotinine, mean (SE), ng/mL^c^	56.60 (1.8)

*Abbreviations*: *NHANES* National Health and Nutrition Examination Survey, *SE* standard error, *PA* physical activity, *OPA* occupation-related PA, *TPA* transportation-related PA, *LTPA* leisure-time PA

^a^Prescription opioids were categorized based on morphine equivalency criteria established by the Centers for Disease Control and Prevention

^b^Sufficient refers to adherence with the 2018 PA guidelines (moderate-intensity PA ≥ 150 min/week)

^c^SI conversion factor: To convert cotinine to nanomoles per liter, multiply by 5.675

### Associations between various domains of PA and prescription opioid use

Table 2 displays the results of multivariable logistic regression analyses between various domains of PA and prescription opioid use. In the unadjusted analysis (model 1), the odds ratio (OR) for prescription opioid use was 0.53 (95% confidence interval [CI], 0.45–0.64), 0.84 (95%CI, 0.70–0.99), 0.68 (95%CI, 0.55–0.86), and 0.46 (95%CI, 0.37–0.58) for those with sufficient total PA, OPA, TPA, and LTPA compared with insufficient PA, respectively. After multivariable adjustment (model3), significantly inverse associations were observed for sufficient total PA (OR,0.68 95%CI [0.56–0.81]), TPA (OR,0.73 95%CI [0.58–0.92]), and LTPA (OR,0.60 95%CI [0.48–0.75]) compared with insufficient PA, but not for OPA (OR,0.93 95%CI [0.79–1.10]). Nevertheless, participants whose total PA met PA guidelines had 1.64 (95%CI, 1.11–2.42) times the odds of reporting short-term use of prescription opioids (Table S2). In contrast, sufficient total PA and LTPA, but not for OPA, were associated with lower odds of long-term use of prescription opioids, regardless of opioid strength. However, sufficient TPA only had a similar inverse association with long-term use of prescription opioid weaker than morphine (Table S3).

**Table 2:** Associations between PA and prescription opioid use among adults in NHANES, 2007 to 2020

	**Odds ratio (95% CI)**		
	**Model 1**^**a**^	**Model 2**^**b**^	**Model 3**^**c**^
**Total physical activity**			
Insufficient	1.00 (Ref)	1.00 (Ref)	1.00 (Ref)
Sufficient	**0.53 (0.45 – 0.64)**	**0.62 (0.52 – 0.73)**	**0.68 (0.56 – 0.81)**
**Occupation-related physical activity**			
Insufficient	1.00 (Ref)	1.00 (Ref)	1.00 (Ref)
Sufficient	**0.84 (0.70 – 0.99)**	0.93 (0.78 – 1.11)	0.93 (0.79 – 1.10)
**Transportation-related physical activity**			
Insufficient	1.00 (Ref)	1.00 (Ref)	1.00 (Ref)
Sufficient	**0.68 (0.55 – 0.86)**	**0.79 (0.63 – 0.99)**	**0.73 (0.58 – 0.92)**
**Leisure-time physical activity**			
Insufficient	1.00 (Ref)	1.00 (Ref)	1.00 (Ref)
Sufficient	**0.46 (0.37 – 0.58)**	**0.51 (0.41 – 0.64)**	**0.60 (0.48 – 0.75)**

*Abbreviations*: *NHANES* National Health and Nutrition Examination Survey, *CI* confidence interval, *PA* physical activity

SI conversion factor: To convert cotinine to nanomoles per liter, multiply by 5.675

^a^Unadjusted

^b^Adjusted for age (continuous), sex (male or female), race/ethnicity (non-Hispanic White, non-Hispanic Black, Mexican American, or other races)

^c^Adjusted for covariates in the model 2 plus education (less than high school, high school, or more than high school), poverty-income ratio (≤ 130%,130%-300%, or > 300%), any insurance (no or yes), alcohol user (never, former, mild, moderate, or heavy), cotinine (continuous), and survey cycle (2007–2008 to 2019–2020)

### Dose–response effect between various domains of PA and prescription opioid use and the additional benefit of PA above or below PA guidelines

Table 3 shows the dose–response effect between different domains of PA and prescription opioid use and assesses the additional benefit of PA above or below PA guidelines. After multivariable adjustment (model2), except for OPA (150–299 vs 0 min/week OR, 0.68 95% CI [0.48–0.96]), participants who reported < 1 time (1–149 min/week), 1–2 times (150–299 min/week), or over two times (≥ 300 min/week) sufficient total PA had a 22% (95% CI 0.64–0.95) to 40% (95% CI 0.50–0.74) lower odds of prescription opioid use compared to 0 min/week. Comparatively, the lower odds were from 36% (95% CI 0.49–0.84) among those with < 1 time sufficient TPA to 27% (95% CI 0.54–0.98) among those with over two times sufficient TPA, and from 26% (95% CI 0.57–0.95) among those with < 1 time sufficient LTPA to 47% (95% CI 0.41–0.70) among those with over two times sufficient LTPA. Interestingly, only participants with 1–2 times (150–299 min/week) sufficient total PA had 3.07 times the odds of short-term use of prescription opioids (95% CI 1.63–5.77) (Table S4). All domains of PA meeting 1–2 times (150 – 299 min/week) sufficient PA levels had the highest inverse associations with long-term use of prescription opioid weaker than morphine. Differently, the highest inverse association was observed for over two times (≥ 300 min/week) sufficient total PA or LTPA, and < 1 time (1–149 min/week) sufficient TPA with long-term use of prescription opioid equivalent to morphine or stronger (Table S5).

**Table 3:** Associations between duration of PA and prescription opioid use among adults in NHANES, 2007 to 2020

	**Odds ratio (95% CI)**				***P*** for trend
	**0 min/week**	**1 – 149 min/week**	**150 – 299 min/week**	** ≥ 300 min/week**	
**Total physical activity**					
Model 1^a^	1.00 (Ref)	0.67 (0.55 – 0.81)	0.55 (0.42 – 0.72)	0.45 (0.37 – 0.54)	** < 0.0001**
Model 2^b^	1.00 (Ref)	0.78 (0.64 – 0.95)	0.69 (0.52 – 0.91)	0.60 (0.50 – 0.74)	** < 0.0001**
**Occupation-related physical activity**					
Model 1^a^	1.00 (Ref)	1.03 (0.80 – 1.33)	0.63 (0.45 – 0.89)	0.87 (0.71 – 1.06)	0.10
Model 2^b^	1.00 (Ref)	1.09 (0.84 – 1.42)	0.68 (0.48 – 0.96)	0.99 (0.81 – 1.20)	0.68
**Transportation-related physical activity**					
Model 1^a^	1.00 (Ref)	0.56 (0.43 – 0.73)	0.58 (0.44 – 0.77)	0.70 (0.52 – 0.94)	** < 0.0001**
Model 2^b^	1.00 (Ref)	0.64 (0.49 – 0.84)	0.66 (0.49 – 0.89)	0.73 (0.54 – 0.98)	**0.001**
**Leisure-time physical activity**					
Model 1^a^	1.00 (Ref)	0.64 (0.49 – 0.82)	0.47 (0.36 – 0.62)	0.39 (0.30 – 0.51)	** < 0.0001**
Model 2^b^	1.00 (Ref)	0.74 (0.57 – 0.95)	0.58 (0.43 – 0.77)	0.53 (0.41 – 0.70)	** < 0.0001**

*Abbreviations*: *NHANES* National Health and Nutrition Examination Survey, *CI* confidence interval, *PA* physical activity

SI conversion factor: To convert cotinine to nanomoles per liter, multiply by 5.675

^a^Unadjusted

^b^Adjusted for age (continuous), sex (male or female), race/ethnicity (non-Hispanic White, non-Hispanic Black, Mexican American, or other races), education (less than high school, high school, or more than high school), poverty-income ratio (≤ 130%,130%-300%, or > 300%), any insurance (no or yes), alcohol user (never, former, mild, moderate, or heavy), cotinine (continuous), and survey cycle (2007–2008 to 2019–2020)

### Stratified analysis and sensitivity analysis

**Fig. 2:**
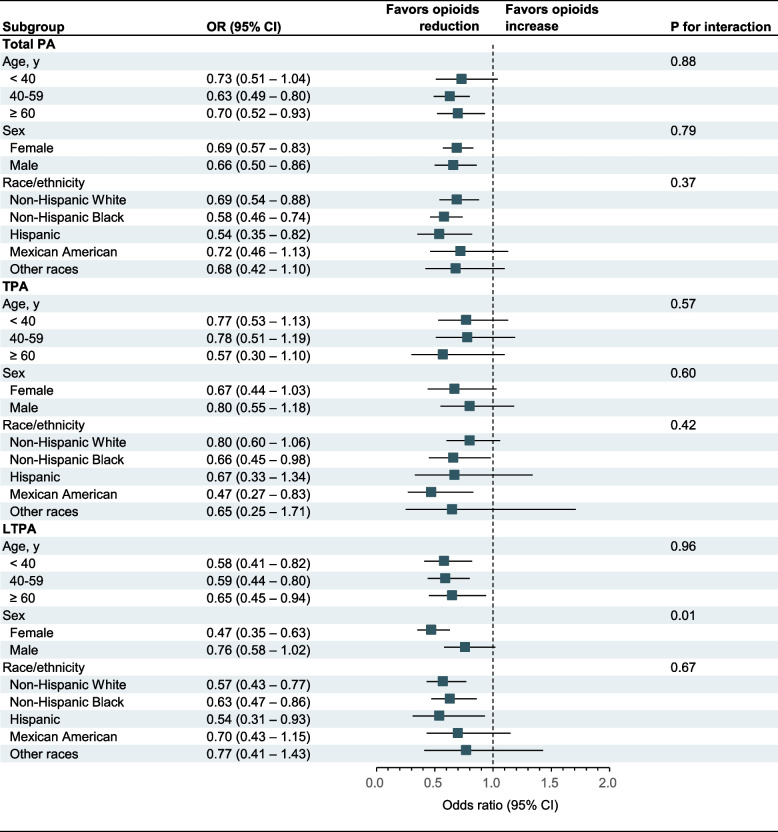
Associations of PA domains with prescription opioid use in subgroups among adults in NHANES, 2007 to 2020. The model was adjusted for age (continuous), sex (male or female), race/ethnicity (non-Hispanic White, non-Hispanic Black, Hispanic, Mexican American, or other races), education (less than high school, high school, or more than high school), poverty-income ratio (≤ 130%,130%-300%, or > 300%), any insurance (no or yes), alcohol user (never, former, mild, moderate, or heavy), cotinine (continuous), and survey cycle (2007-2008 to 2019-2020). The strata variable was not included in the adjustment when stratifying by itself

In stratified analysis, only sex (p for interaction = 0.01) was observed to significantly modify the relationship between LTPA and prescription opioid use (Fig. 2). For the subgroup of female, the OR for prescription opioid use was 0.47 (95% CI, 0.35–0.63) for those with sufficient PA compared with insufficient PA, and in the subgroup of male, the OR for prescription opioid use was 0.76 (95% CI, 0.58–1.02) for those with sufficient PA compared with insufficient PA. Likewise, no modification factors were found in the short-term and long-term use of prescription opioids (Table S6 and S7). In sensitivity analysis, the associations were typically robust when further excluding participants who took benzodiazepines and SSRIs antidepressants (Table S8–10), reanalyzing by prescription opioid use < 30 days and ≥ 30 days (Table S11 and S12), and multiple imputation data analysis (Table S14–S17). Nevertheless, we also found an inverse association of sufficient TPA (OR, 0.57 95% CI [0.35–0.92]) with short-term use of prescription opioids after multiple imputation. The E-value indicated that the observed OR in favor of total PA could be explained away by an unmeasured confounder that was associated with sufficient total PA and prescription opioid use by an OR at least 2.30-fold each. Similarly, the E-values for sufficient TPA and LTPA were 2.08-fold and 2.72-fold, respectively (Table S13). The E-values for short-term and long-term use of prescription opioids were also seen in Table S13.

## Discussion

In this study, we discovered significantly inverse associations between sufficient total PA, TPA, or LTPA and prescription opioid use, with a dose-dependent effect. However, we found that 1–2 times (150–299 min/week) sufficient total PA was more likely to be associated with short-term use of prescription opioids. For long-term use of prescription opioid weaker than morphine, the highest inverse association was observed for 1–2 times (150–299 min/week) sufficient PA, regardless of PA domains. Comparatively, < 1 times (1–149 min/week) sufficient TPA, and over 2 times (≥ 300 min/week) sufficient total PA and LTPA exhibited the highest inverse associations with long-term use of prescription opioid equal to morphine or stronger.

Existing researches on the association of PA with prescription opioid use have been mixed. Previous studies had reported opioid use was associated with a lack of PA during leisure time [38, 39]. A Danish study found that drug intake decreased during exercise in 38 addicted individuals, and long-term follow-up supported this finding [40]. A survey conducted among residents of New York City revealed that individuals without PA were more likely to use inappropriate opioids [41]. For individuals with osteoarthritis, exercise therapy resulted in a 36% relative reduction in the proportion of opioid user [42], while a study in the same region suggested that the decrease in oral morphine equivalents was primarily attributed to regulatory actions targeting opioid prescription [43]. Preclinical studies had demonstrated that exercise could reduce self-administration of morphine [44]. Nevertheless, some studies did not support our main argument. Carpenter RW et al. argued that higher occasion-level PA was associated with an increased likelihood of opioid use in patients with chronic low back pain, irrespective of their pain status [30]. Notwithstanding, the results need to be interpreted with caution due to the limited sample. Andrews NE et al. reported that overactivity was linked to opioid dependence, with higher levels of overactivity being more likely to use prescription opioids [45, 46]. Comparing of these studies is challenging due to variations in PA assessment and study design. Furthermore, variations in the study populations, such as the exclusive analysis of lower back pain or joint pain in some studies, may introduce biases in the research conclusions.

Previous studies have found that there are significant differences in the effects of LTPA and OPA on health. LTPA often has a positive effect on related diseases including pain and cancer, while OPA is considered to have a negative effect [20, 21, 23–25]. This effect is further supported by our finding that participants with higher LTPA might have lower odds of reporting prescription opioid use. Nevertheless, no association was found between OPA and prescription opioid use, and we speculate whether this is due to confusion caused by different types of job. Given more sedentary jobs and fewer physically demanding jobs, the areas of overload and exertion tend to be confined to the hands, neck and shoulders, where the pain caused doesn't make prescription opioid use a first choice. In secondary analysis, we discovered that 1–2 times (150–299 min/week) sufficient total PA was a risk factor for short-term use of prescription opioids. This might be due to participants engaging in inappropriate PA, which raised the likelihood of sudden injuries, trauma, and surgeries, leading to prescribe short-term opioids for acute or subacute pain.

The potential mechanisms underlying the inverse association of PA and prescription opioid use remain unclear. PA can reduce cravings for addictive substances by releasing endogenous opiates and endocannabinoids [47, 48]. Studies have indicated that exercise has a protective effect on dopamine system homeostasis by increasing dopamine levels and balancing the D1 receptor (D1R) and D2 receptor (D2R) ratio to normalize the reward system [49, 50]. PA may enhance inhibitory control, stress management, and emotion regulation in the medial prefrontal cortex (mPFC) through upregulating neurotrophic factors such as brain-derived neurotrophic factor (BDNF), thereby reducing drug abuse and addiction [51–53]. Additionally, evidence suggests that exercise may modulate the dysfunctions of oxytocin and hypothalamic–pituitary–adrenal (HPA) axis caused by opioids, resulting in a reduction in anxiety and stress responses [51, 54–56].

To our knowledge, this study is the first to examine the relationship between different domains of PA and prescription opioid use. In contrast to prior research, our study is based on a nationally representative US adult population, which facilitates the extrapolation of findings. Moreover, we also explored the impact of PA on prescription opioid use in terms of the duration and strength of opioids. Our study also has some limitations. *Firstly, the nature of cross-sectional design presents challenges in establishing causality*, prospective studies are necessary to investigate this association in the future. Then, PA was evaluated through self-report questionnaires, which is susceptible to recall bias. Thirdly, as NHANES did not record the dosage of prescription opioid use, we had to rely on the morphine equivalency criteria created by the Centers for Disease Control and Prevention (CDC) to evaluate the strength of opioids. Fourthly, this study was limited by the small sample size of short-term use of prescription opioid weaker than morphine, therefore did not compare the effects of PA on short-term use of different strength of opioids. Finally, despite the robust overall associations indicated by the E-values, the residual or unknown confounders can not be completely ruled out.

## Conclusions

In conclusion, we found sufficient total PA, TPA, and LTPA were inversely associated with prescription opioid use and varied depending on the duration and strength of prescription opioid use. Significantly, 1–2 times (150—299 min/week) sufficient total PA might be a potential risk factor for short-term use of prescription opioids. These findings underscore the rational allocation of different domains of PA may be useful to reduce prescription opioid use.

### Supplementary Information

**Supplementary Material 1.**

## Data Availability

The data analyzed during the current study are publicly available at the NHANES website http://www.cdc.gov/nchs/nhanes.htm.

## Abbreviations

PA: Physical activity
OPA: Occupation-related physical activity
TPA: Transportation-related physical activity
LTPA: Leisure-time physical activity
NCDs: Chronic non-communicable diseases
NHANES: National Health and Nutrition Examination Survey
GPAQ: Global Physical Activity Questionnaire
MMT: Methadone maintenance treatment
NCHS: National Center of Health Statistics
PIR: Poverty-income ratio
SSRIs: Selective serotonin reuptake inhibitors
OR: Odds ratio
CI: Confidence interval
D1R: D1 receptor
D2R: D2 receptor
mPFC: Medial prefrontal cortex
BDNF: Brain-derived neurotrophic factor
HPA: Hypothalamic-pituitary-adrenal
CDC: Centers for Disease Control and Prevention
