# Supplementary material for: Analysis of physical activity and prescription opioid use among US adults: a cross-sectional study
Source: BMC Public Health. 2024 Mar 5;24:698. doi: 10.1186/s12889-024-18220-7 (PMC10913271; doi:10.1186/s12889-024-18220-7)
Supplement: Supplementary file 1 — Supplementary Material 1. [file 12889_2024_18220_MOESM1_ESM.docx]

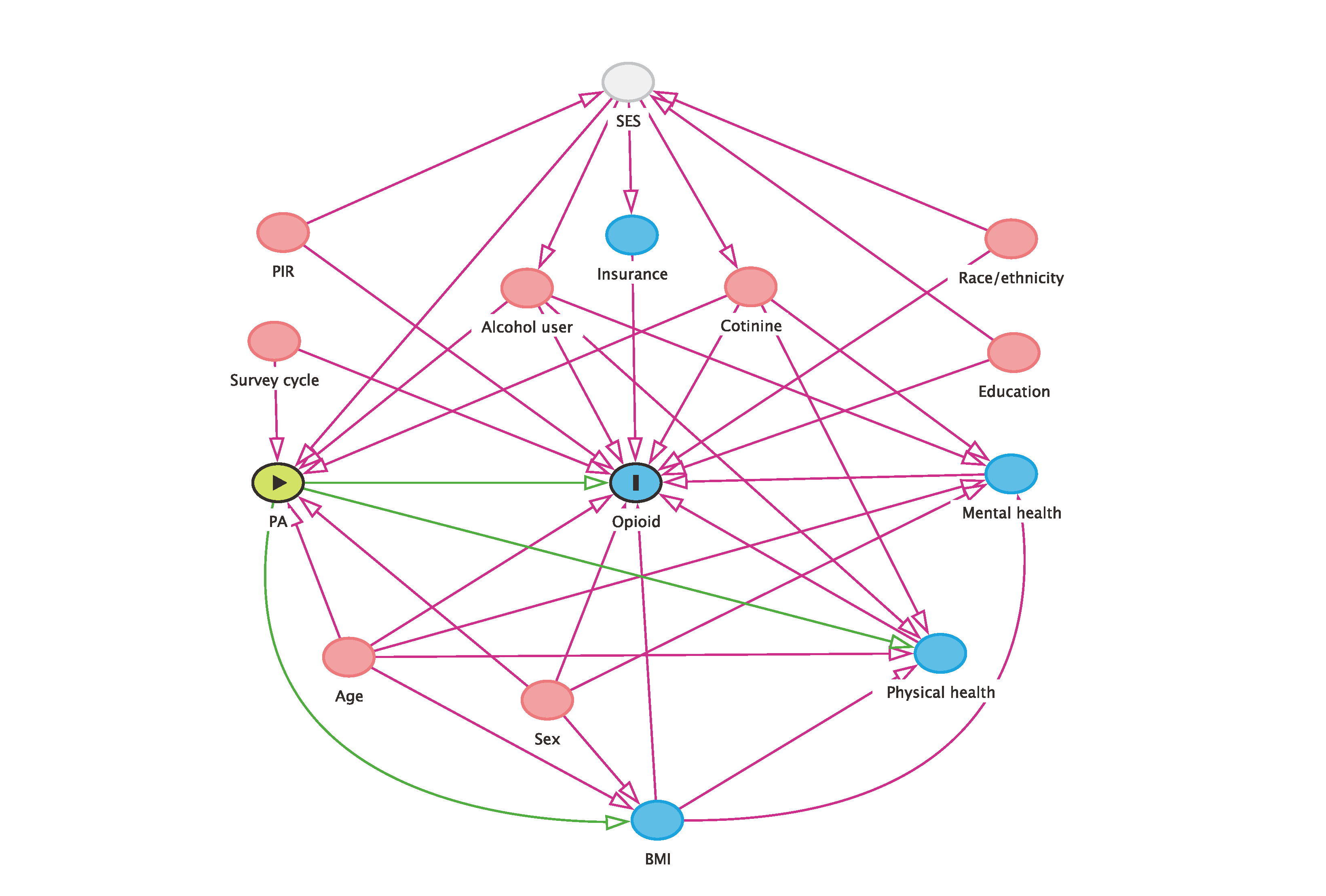


**Figure S1.** **Directed acyclic graph of the effect of physical activity on prescription opioid use**

Abbreviations: PA, physical activity; BMI, body mass index; PIR, poverty-income ratio; SES, socio-economic status. (Available at http://dagitty.net/)

**Table S1.** **Classification of strength of prescription opioids^*^**

| **Weaker than morphine** |
| --- |
| acetaminophen; butalbital; caffeine; codeine |
| acetaminophen; caffeine; dihydrocodeine |
| acetaminophen; propoxyphene |
| acetaminophen; pentazocine |
| acetaminophen; tramadol |
| acetaminophen; codeine |
| aspirin; butalbital; caffeine; codeine |
| anhydrous calcium iodide; codeine |
| chlorpheniramine; dihydrocodeine; phenylephrine |
| chlorpheniramine; codeine; pseudoephedrine |
| codeine; guaifenesin; pseudoephedrine |
| codeine; phenylephrine; promethazine |
| codeine; guaifenesin |
| codeine; promethazine |
| codeine |
| dihydrocodeine; guaifenesin; pseudoephedrine |
| meperidine; promethazine |
| meperidine |
| naloxone; pentazocine |
| propoxyphene |
| pentazocine |
| tramadol |
| **Equivalent to morphine** |
| acetaminophen; caffeine; chlorpheniramine; hydrocodone; phenylephrine |
| acetaminophen; hydrocodone |
| aspirin; hydrocodone |
| brompheniramine; hydrocodone; phenylephrine |
| brompheniramine; hydrocodone; pseudoephedrine |
| belladonna; opium |
| chlorpheniramine; hydrocodone; pseudoephedrine |
| chlorpheniramine; hydrocodone; phenylephrine |
| chlorpheniramine; hydrocodone |
| dexbrompheniramine; hydrocodone; phenylephrine |
| diphenhydramine; hydrocodone; phenylephrine |
| guaifenesin; hydrocodone; pseudoephedrine |
| guaifenesin; hydrocodone |
| hydrocodone; pseudoephedrine; triprolidine |
| hydrocodone; phenylephrine; pyrilamine |
| hydrocodone; phenylpropanolamine |
| hydrocodone; phenylephrine |
| hydrocodone; ibuprofen |
| hydrocodone |
| homatropine; hydrocodone |
| morphine |
| opium |
| tapentadol |
| **Stronger than morphine** |
| acetaminophen; oxycodone |
| aspirin; oxycodone |
| fentanyl |
| hydromorphone |
| oxymorphone |
| oxycodone |

^*^ Prescription opioids were categorized based on morphine equivalency criteria established by the Centers for Disease Control and Prevention. In addition, those commonly used to treat opioid addiction or opioid withdrawal (including methadone, buprenorphine, and buprenorphine; naloxone) were excluded.

**Table S2. Associations between PA and short-term use of prescription opioids among adults in NHANES, 2007 to 2020***

|  | **Odds ratio (95% CI)** | | |
| --- | --- | --- | --- |
|  | **Model 1^†^** | **Model 2^‡^** | **Model 3^§^** |
| **Total PA** |  |  |  |
| Insufficient | 1.00 (Ref) | 1.00 (Ref) | 1.00 (Ref) |
| Sufficient | **1.82 (1.26 – 2.63)** | **1.59 (1.08 – 2.34)** | **1.64 (1.11 – 2.42)** |
| **OPA** |  |  |  |
| Insufficient | 1.00 (Ref) | 1.00 (Ref) | 1.00 (Ref) |
| Sufficient | 1.23 (0.85 – 1.78) | 1.12 (0.76 – 1.65) | 1.07 (0.71 – 1.59) |
| **TPA** |  |  |  |
| Insufficient | 1.00 (Ref) | 1.00 (Ref) | 1.00 (Ref) |
| Sufficient | 0.79 (0.48 – 1.30) | 0.73 (0.44 – 1.21) | 0.68 (0.41 – 1.15) |
| **LTPA** |  |  |  |
| Insufficient | 1.00 (Ref) | 1.00 (Ref) | 1.00 (Ref) |
| Sufficient | 1.07 (0.74 – 1.55) | 0.94 (0.64 – 1.38) | 1.02 (0.67 – 1.57) |

Abbreviations: NHANES, National Health and Nutrition Examination Survey; CI, confidence interval; PA, physical activity; OPA, occupation-related PA; TPA, transportation-related PA; LTPA, leisure-time PA.

SI conversion factor: To convert cotinine to nanomoles per liter, multiply by 5.675.

^*^ No opioid use was used as comparison.

^†^ Unadjusted.

^‡^ Adjusted for age (continuous), sex (male or female), race/ethnicity (non-Hispanic White, non-Hispanic Black, Mexican American, or other races).

^§^ Adjusted for covariates in the model 2 plus education (less than high school, high school, or more than high school), poverty-income ratio (≤130%,130%-300%, or >300%), any insurance (no or yes), alcohol user (never, former, mild, moderate, or heavy), cotinine (continuous), and survey cycle (2007-2008 to 2019-2020).

**Table S3. Associations between PA and long-term use of prescription opioids among adults in NHANES, 2007 to 2020***

|  | **Odds ratio (95% CI)** | | | | | |
| --- | --- | --- | --- | --- | --- | --- |
|  | **Weaker than morphine** | | | **Equivalent to morphine or stronger** | | |
|  | **Model 1^†^** | **Model 2^‡^** | **Model 3^§^** | **Model 1^†^** | **Model 2^‡^** | **Model 3^§^** |
| **Total PA** |  |  |  |  |  |  |
| Insufficient | 1.00 (Ref) | 1.00 (Ref) | 1.00 (Ref) | 1.00 (Ref) | 1.00 (Ref) | 1.00 (Ref) |
| Sufficient | **0.42 (0.31 – 0.57)** | **0.57 (0.41 – 0.78)** | **0.61 (0.44 – 0.85)** | **0.44 (0.36 – 0.53)** | **0.51 (0.42 – 0.62)** | **0.57 (0.47 – 0.70)** |
| **OPA** |  |  |  |  |  |  |
| Insufficient | 1.00 (Ref) | 1.00 (Ref) | 1.00 (Ref) | 1.00 (Ref) | 1.00 (Ref) | 1.00 (Ref) |
| Sufficient | 0.87 (0.62 – 1.21) | 1.09 (0.78 – 1.54) | 1.11 (0.79 – 1.55) | **0.73 (0.61 – 0.88)** | 0.83 (0.68 – 1.01) | 0.83 (0.68 – 1.01) |
| **TPA** |  |  |  |  |  |  |
| Insufficient | 1.00 (Ref) | 1.00 (Ref) | 1.00 (Ref) | 1.00 (Ref) | 1.00 (Ref) | 1.00 (Ref) |
| Sufficient | **0.46 (0.29 – 0.73)** | **0.55 (0.35 – 0.88)** | **0.55 (0.35 – 0.86)** | 0.76 (0.57 – 1.00) | 0.91 (0.69 – 1.21) | 0.84 (0.63 – 1.12) |
| **LTPA** |  |  |  |  |  |  |
| Insufficient | 1.00 (Ref) | 1.00 (Ref) | 1.00 (Ref) | 1.00 (Ref) | 1.00 (Ref) | 1.00 (Ref) |
| Sufficient | **0.34 (0.22 – 0.53)** | **0.43 (0.28 – 0.66)** | **0.47 (0.31 – 0.72)** | **0.39 (0.30 – 0.50)** | **0.44 (0.34 – 0.56)** | **0.52 (0.41 – 0.68)** |

Abbreviations: NHANES, National Health and Nutrition Examination Survey; CI, confidence interval; PA, physical activity; OPA, occupation-related PA; TPA, transportation-related PA; LTPA, leisure-time PA.

SI conversion factor: To convert cotinine to nanomoles per liter, multiply by 5.675.

^*^ No opioid use was used as comparison.

^†^ Unadjusted.

^‡^ Adjusted for age (continuous), sex (male or female), race/ethnicity (non-Hispanic White, non-Hispanic Black, Mexican American, or other races).

^§^ Adjusted for covariates in the model 2 plus education (less than high school, high school, or more than high school), poverty-income ratio (≤130%,130%-300%, or >300%), any insurance (no or yes), alcohol user (never, former, mild, moderate, or heavy), cotinine (continuous), and survey cycle (2007-2008 to 2019-2020).

**Table S4. Associations between duration of PA and short-term use of prescription opioids among adults in NHANES, 2007 to 2020***

|  | **Odds ratio (95% CI)** | | | | **P for trend** |
| --- | --- | --- | --- | --- | --- |
|  | **0 min/week** | **1 – 149 min/week** | **150 – 299 min/week** | **≥ 300 min/week** |  |
| **Total PA** |  |  |  |  |  |
| Model 1^†^ | 1.00 (Ref) | 0.90 (0.50 – 1.63) | 3.09 (1.68 – 5.69) | 1.52 (0.97 – 2.37) | **0.04** |
| Model 2^‡^ | 1.00 (Ref) | 0.89 (0.49 – 1.63) | 3.07 (1.63 – 5.77) | 1.29 (0.80 – 2.07) | 0.25 |
| **OPA** |  |  |  |  |  |
| Model 1^†^ | 1.00 (Ref) | 1.72 (0.82 – 3.59) | 0.67 (0.30 – 1.50) | 1.45 (1.00 – 2.10) | 0.09 |
| Model 2^‡^ | 1.00 (Ref) | 1.78 (0.86 – 3.69) | 0.67 (0.30 – 1.52) | 1.26 (0.84 – 1.87) | 0.38 |
| **TPA** |  |  |  |  |  |
| Model 1^†^ | 1.00 (Ref) | 1.13 (0.64 – 2.01) | 0.47 (0.19 – 1.15) | 1.07 (0.59 – 1.92) | 0.74 |
| Model 2^‡^ | 1.00 (Ref) | 1.05 (0.57 – 1.93) | 0.41 (0.16 – 1.03) | 0.90 (0.49 – 1.65) | 0.38 |
| **LTPA** |  |  |  |  |  |
| Model 1^†^ | 1.00 (Ref) | 1.35 (0.76 – 2.39) | 1.48 (0.84 – 2.63) | 1.03 (0.65 – 1.63) | 0.72 |
| Model 2^‡^ | 1.00 (Ref) | 1.37 (0.76 – 2.46) | 1.51 (0.79 – 2.88) | 0.97 (0.59 – 1.61) | 0.94 |

Abbreviations: NHANES, National Health and Nutrition Examination Survey; CI, confidence interval; PA, physical activity; OPA, occupation-related PA; TPA, transportation-related PA; LTPA, leisure-time PA.

SI conversion factor: To convert cotinine to nanomoles per liter, multiply by 5.675.

^*^ No opioid use was used as comparison.

^†^ Unadjusted.

^‡^ Adjusted for age (continuous), sex (male or female), race/ethnicity (non-Hispanic White, non-Hispanic Black, Mexican American, or other races), education (less than high school, high school, or more than high school), poverty-income ratio (≤130%,130%-300%, or >300%), any insurance (no or yes), alcohol user (never, former, mild, moderate, or heavy), cotinine (continuous), and survey cycle (2007-2008 to 2019-2020).

**Table S5. Associations between duration of PA and long-term use of prescription opioids among adults in NHANES, 2007 to 2020***

|  | **Odds ratio (95% CI)** | | | | | | | | | |
| --- | --- | --- | --- | --- | --- | --- | --- | --- | --- | --- |
|  | **Weaker than morphine** | | | | **P for trend** | **Equivalent to morphine or stronger** | | | | **P for trend** |
|  | **0**  **min/week** | **1 – 149**  **min/week** | **150 – 299**  **min/week** | **≥ 300**  **min/week** |  | **0**  **min/week** | **1 – 149**  **min/week** | **150 – 299**  **min/week** | **≥ 300**  **min/week** |  |
| **Total PA** |  |  |  |  |  |  |  |  |  |  |
| Model 1^†^ | 1.00 (Ref) | 0.76 (0.54 – 1.07) | 0.28 (0.17 –0.46) | 0.40 (0.28 –0.56) | **<0.0001** | 1.00 (Ref) | 0.60 (0.47 –0.77) | 0.42 (0.31 –0.56) | 0.36 (0.29 –0.45) | **<0.0001** |
| Model 2^‡^ | 1.00 (Ref) | 0.93 (0.66 –1.33) | 0.38 (0.22 –0.64) | 0.65 (0.45 –0.94) | **0.01** | 1.00 (Ref) | 0.72 (0.56 –0.93) | 0.53 (0.39 –0.72) | 0.51 (0.41 –0.64) | **<0.0001** |
| **OPA** |  |  |  |  |  |  |  |  |  |  |
| Model 1^†^ | 1.00 (Ref) | 0.92 (0.60 –1.41) | 0.35 (0.15 –0.81) | 0.93 (0.65 –1.34) | 0.55 | 1.00 (Ref) | 0.93 (0.66 –1.32) | 0.74 (0.49 –1.14) | 0.72 (0.57 –0.91) | **0.003** |
| Model 2^‡^ | 1.00 (Ref) | 0.97 (0.63 –1.50) | 0.39 (0.17 –0.93) | 1.24 (0.86 –1.78) | 0.39 | 1.00 (Ref) | 0.98 (0.68 –1.41) | 0.81 (0.53 –1.25) | 0.83 (0.65 –1.07) | 0.11 |
| **TPA** |  |  |  |  |  |  |  |  |  |  |
| Model 1^†^ | 1.00 (Ref) | 0.40 (0.26 –0.62) | 0.34 (0.16 –0.71) | 0.49 (0.27 –0.88) | **<0.001** | 1.00 (Ref) | 0.49 (0.34 –0.72) | 0.72 (0.52 –1.01) | 0.70 (0.47 –1.03) | **0.004** |
| Model 2^‡^ | 1.00 (Ref) | 0.50 (0.32 –0.76) | 0.41 (0.20 –0.87) | 0.58 (0.33 –1.04) | **0.003** | 1.00 (Ref) | 0.57 (0.39 –0.84) | 0.86 (0.60 –1.22) | 0.74 (0.49 –1.12) | **0.04** |
| **LTPA** |  |  |  |  |  |  |  |  |  |  |
| Model 1^†^ | 1.00 (Ref) | 0.59 (0.40 –0.88) | 0.23 (0.16 –0.33) | 0.34 (0.19 –0.58) | **<0.0001** | 1.00 (Ref) | 0.54 (0.42 –0.69) | 0.41 (0.30 –0.56) | 0.30 (0.22 –0.42) | **<0.0001** |
| Model 2^‡^ | 1.00 (Ref) | 0.70 (0.46 –1.06) | 0.29 (0.19 –0.43) | 0.51 (0.30 –0.87) | **0.001** | 1.00 (Ref) | 0.64 (0.49 –0.82) | 0.52 (0.38 –0.72) | 0.44 (0.32 –0.60) | **<0.0001** |

Abbreviations: NHANES, National Health and Nutrition Examination Survey; CI, confidence interval; PA, physical activity; OPA, occupation-related PA; TPA, transportation-related PA; LTPA, leisure-time PA.

SI conversion factor: To convert cotinine to nanomoles per liter, multiply by 5.675.

^*^ No opioid use was used as comparison.

^†^ Unadjusted.

^‡^ Adjusted for age (continuous), sex (male or female), race/ethnicity (non-Hispanic White, non-Hispanic Black, Mexican American, or other races), education (less than high school, high school, or more than high school), poverty-income ratio (≤130%,130%-300%, or >300%), any insurance (no or yes), alcohol user (never, former, mild, moderate, or heavy), cotinine (continuous), and survey cycle (2007-2008 to 2019-2020).

**Table S6.** **Associations of total PA with short-term use of prescription opioids in subgroups among adults in NHANES, 2007 to 2020^*^**

|  | **Odds ratio (95% CI)^†^** | | |
| --- | --- | --- | --- |
|  | **Insufficient** | **Sufficient** | **P for interaction** |
| Age, y |  |  | 0.43 |
| 20-39 | 1.00 (Ref) | 1.40 (0.90 – 2.17) |  |
| 40-59 | 1.00 (Ref) | 2.15 (1.09 – 4.22) |  |
| ≥ 60 | 1.00 (Ref) | 1.72 (0.67 – 4.45) |  |
| Sex |  |  | 0.90 |
| Female | 1.00 (Ref) | 1.49 (0.95 – 2.35) |  |
| Male | 1.00 (Ref) | 1.77 (0.94 – 3.34) |  |
| Race/ethnicity |  |  | 0.12 |
| Non-Hispanic White | 1.00 (Ref) | 2.04 (1.16 – 3.61) |  |
| Non-Hispanic Black | 1.00 (Ref) | 1.84 (0.89 – 3.78) |  |
| Hispanic | 1.00 (Ref) | 0.38 (0.18 – 0.84) |  |
| Mexican American | 1.00 (Ref) | 1.92 (0.84 – 4.42) |  |
| Other races | 1.00 (Ref) | 0.69 (0.24 – 1.97) |  |

Abbreviations: NHANES, National Health and Nutrition Examination Survey; CI, confidence interval; PA, physical activity; OPA, occupation-related PA; TPA, transportation-related PA; LTPA, leisure-time PA.

SI conversion factor: To convert cotinine to nanomoles per liter, multiply by 5.675.

^*^ No opioid use was used as comparison.

^†^ Adjusted for age (continuous), sex (male or female), race/ethnicity (non-Hispanic White, non-Hispanic Black, Mexican American, or other races), education (less than high school, high school, or more than high school), poverty-income ratio (≤130%,130%-300%, or >300%), any insurance (no or yes), alcohol user (never, former, mild, moderate, or heavy), cotinine (continuous), and survey cycle (2007-2008 to 2019-2020). The strata variable was not included in the adjustment when stratifying by itself.

**Table S7.** **Associations of PA with long-term use of prescription opioids in subgroups among adults in NHANES, 2007 to 2020^*^**

|  | **Odds ratio (95% CI)^†^** | | | | | |
| --- | --- | --- | --- | --- | --- | --- |
|  | **Weaker than morphine** | | **P for interaction** | **Equivalent to morphine or stronger** | | **P for interaction** |
|  | **Insufficient** | **Sufficient** |  | **Insufficient** | **Sufficient** |  |
| **Total PA** |  |  |  |  |  |  |
| Age, y |  |  | 0.65 |  |  | 0.65 |
| 20-39 | 1.00 (Ref) | 0.41 (0.15 – 1.13) |  | 1.00 (Ref) | 0.54 (0.34 – 0.85) |  |
| 40-59 | 1.00 (Ref) | 0.62 (0.40 – 0.96) |  | 1.00 (Ref) | 0.53 (0.39 – 0.72) |  |
| ≥ 60 | 1.00 (Ref) | 0.64 (0.44 – 0.93) |  | 1.00 (Ref) | 0.66 (0.46 – 0.96) |  |
| Sex |  |  | 0.41 |  |  | 0.27 |
| Female | 1.00 (Ref) | 0.56 (0.38 – 0.83) |  | 1.00 (Ref) | 0.64 (0.50 – 0.81) |  |
| Male | 1.00 (Ref) | 0.70 (0.41 – 1.18) |  | 1.00 (Ref) | 0.50 (0.36 – 0.67) |  |
| Race/ethnicity |  |  | 0.23 |  |  | 0.78 |
| Non-Hispanic White | 1.00 (Ref) | 0.64 (0.41 – 1.01) |  | 1.00 (Ref) | 0.58 (0.45 – 0.74) |  |
| Non-Hispanic Black | 1.00 (Ref) | 0.46 (0.28 – 0.76) |  | 1.00 (Ref) | 0.49 (0.35 – 0.67) |  |
| Hispanic | 1.00 (Ref) | 0.59 (0.27– 1.29) |  | 1.00 (Ref) | 0.60(0.32 – 1.13) |  |
| Mexican American | 1.00 (Ref) | 0.45 (0.18 – 1.08) |  | 1.00 (Ref) | 0.59 (0.33 – 1.06) |  |
| Other races | 1.00 (Ref) | 0.90 (0.38 – 2.14) |  | 1.00 (Ref) | 0.54 (0.25 – 1.14) |  |
| **TPA** |  |  |  |  |  |  |
| Age, y |  |  | 0.51 |  |  | – |
| 20-39 | 1.00 (Ref) | 0.80 (0.30 – 2.14) |  | – | – |  |
| 40-59 | 1.00 (Ref) | 0.59 (0.29 – 1.20) |  | – | – |  |
| ≥ 60 | 1.00 (Ref) | 0.35 (0.19 – 0.65) |  | – | – |  |
| Sex |  |  | 0.97 |  |  | – |
| Female | 1.00 (Ref) | 0.51 (0.27 – 0.96) |  | – | – |  |
| Male | 1.00 (Ref) | 0.59 (0.31 – 1.10) |  | – | – |  |
| Race/ethnicity |  |  | 0.46 |  |  | – |
| Non-Hispanic White | 1.00 (Ref) | 0.47 (0.24 – 0.93) |  | – | – |  |
| Non-Hispanic Black | 1.00 (Ref) | 0.61 (0.28 – 1.33) |  | – | – |  |
| Hispanic | 1.00 (Ref) | 0.60 (0.24 – 1.51) |  |  |  |  |
| Mexican American | 1.00 (Ref) | 0.34 (0.06 – 1.74) |  | – | – |  |
| Other races | 1.00 (Ref) | 1.26 (0.32 – 4.97) |  | – | – |  |
| **LTPA** |  |  |  |  |  |  |
| Age, y |  |  | 0.77 |  |  | 0.77 |
| 20-39 | 1.00 (Ref) | 0.42 (0.17 – 1.02) |  | 1.00 (Ref) | 0.50 (0.31 – 0.80) |  |
| 40-59 | 1.00 (Ref) | 0.41 (0.20 – 0.84) |  | 1.00 (Ref) | 0.60 (0.41 – 0.86) |  |
| ≥ 60 | 1.00 (Ref) | 0.64 (0.38 – 1.06) |  | 1.00 (Ref) | 0.47 (0.31 – 0.71) |  |
| Sex |  |  | 0.20 |  |  | 0.29 |
| Female | 1.00 (Ref) | 0.37 (0.22 – 0.60) |  | 1.00 (Ref) | 0.46 (0.33 – 0.66) |  |
| Male | 1.00 (Ref) | 0.59 (0.32 – 1.07) |  | 1.00 (Ref) | 0.60 (0.41 – 0.88) |  |
| Race/ethnicity |  |  | 0.99 |  |  | 0.77 |
| Non-Hispanic White | 1.00 (Ref) | 0.45 (0.25 – 0.80) |  | 1.00 (Ref) | 0.52 (0.38 – 0.70) |  |
| Non-Hispanic Black | 1.00 (Ref) | 0.58 (0.35 – 0.95) |  | 1.00 (Ref) | 0.52 (0.35 – 0.77) |  |
| Hispanic | 1.00 (Ref) | 0.78(0.38 –1.61) |  | 1.00 (Ref) | 0.37 (0.12 – 1.14) |  |
| Mexican American | 1.00 (Ref) | 0.54 (0.22 – 1.35) |  | 1.00 (Ref) | 0.63 (0.32 – 1.22 ) |  |
| Other races | 1.00 (Ref) | 0.41 (0.14 – 1.18) |  | 1.00 (Ref) | 0.72 (0.28 – 1.87) |  |

Abbreviations: NHANES, National Health and Nutrition Examination Survey; CI, confidence interval; PA, physical activity; OPA, occupation-related PA; TPA, transportation-related PA; LTPA, leisure-time PA.

SI conversion factor: To convert cotinine to nanomoles per liter, multiply by 5.675.

^*^ No opioid use was used as comparison.

^†^ Adjusted for age (continuous), sex (male or female), race/ethnicity (non-Hispanic White, non-Hispanic Black, Mexican American, or other races), education (less than high school, high school, or more than high school), poverty-income ratio (≤130%,130%-300%, or >300%), any insurance (no or yes), alcohol user (never, former, mild, moderate, or heavy), cotinine (continuous), and survey cycle (2007-2008 to 2019-2020). The strata variable was not included in the adjustment when stratifying by itself.

**Table S8. Associations between PA and prescription opioid use among adults without taking benzodiazepines or SSRIs** **antidepressants in NHANES, 2007 to 2020**

|  | **Odds ratio (95% CI)** | | |
| --- | --- | --- | --- |
|  | **Model 1*** | **Model 2^†^** | **Model 3^‡^** |
| **Total PA** |  |  |  |
| Insufficient | 1.00 (Ref) | 1.00 (Ref) | 1.00 (Ref) |
| Sufficient | **0.60 (0.49 – 0.73)** | **0.69 (0.56 – 0.84)** | **0.74 (0.61 – 0.91)** |
| **OPA** |  |  |  |
| Insufficient | 1.00 (Ref) | 1.00 (Ref) | 1.00 (Ref) |
| Sufficient | 0.90 (0.74 – 1.09) | 0.99 (0.82 – 1.20) | 0.98 (0.81 – 1.18) |
| **TPA** |  |  |  |
| Insufficient | 1.00 (Ref) | 1.00 (Ref) | 1.00 (Ref) |
| Sufficient | **0.72 (0.57 – 0.92)** | 0.83 (0.65 – 1.05) | **0.77 (0.60 – 0.98)** |
| **LTPA** |  |  |  |
| Insufficient | 1.00 (Ref) | 1.00 (Ref) | 1.00 (Ref) |
| Sufficient | **0.51 (0.40 – 0.66)** | **0.57 (0.44 – 0.73)** | **0.65 (0.50 – 0.84)** |

Abbreviations: NHANES, National Health and Nutrition Examination Survey; CI, confidence interval; SSRIs, selective serotonin reuptake inhibitor(s); PA, physical activity; OPA, occupation-related PA; TPA, transportation-related PA; LTPA, leisure-time PA.

SI conversion factor: To convert cotinine to nanomoles per liter, multiply by 5.675.

^*^ Unadjusted.

^†^ Adjusted for age (continuous), sex (male or female), race/ethnicity (non-Hispanic White, non-Hispanic Black, Mexican American, or other races).

^‡^ Adjusted for covariates in the model 2 plus education (less than high school, high school, or more than high school), poverty-income ratio (≤130%,130%-300%, or >300%), any insurance (no or yes), alcohol user (never, former, mild, moderate, or heavy), cotinine (continuous), and survey cycle (2007-2008 to 2019-2020).

**Table S9. Associations between PA and short-term use of prescription opioids among adults without taking benzodiazepines or SSRIs** **antidepressants in NHANES, 2007 to 2020^*^**

|  | **Odds ratio (95% CI)** | | |
| --- | --- | --- | --- |
|  | **Model 1^†^** | **Model 2^‡^** | **Model 3^§^** |
| **Total PA** |  |  |  |
| Insufficient | 1.00 (Ref) | 1.00 (Ref) | 1.00 (Ref) |
| Sufficient | **1.75 (1.19 – 2.56)** | **1.55 (1.03 – 2.31)** | **1.60 (1.07 – 2.39)** |
| **OPA** |  |  |  |
| Insufficient | 1.00 (Ref) | 1.00 (Ref) | 1.00 (Ref) |
| Sufficient | 1.19 (0.82 – 1.72) | 1.10 (0.75 – 1.61) | 1.04 (0.69 – 1.55) |
| **TPA** |  |  |  |
| Insufficient | 1.00 (Ref) | 1.00 (Ref) | 1.00 (Ref) |
| Sufficient | 0.78 (0.47 – 1.29) | 0.73 (0.44 – 1.22) | 0.68 (0.40 – 1.16) |
| **LTPA** |  |  |  |
| Insufficient | 1.00 (Ref) | 1.00 (Ref) | 1.00 (Ref) |
| Sufficient | 1.05 (0.72 – 1.53) | 0.94 (0.64 – 1.38) | 1.03 (0.67 – 1.58) |

Abbreviations: NHANES, National Health and Nutrition Examination Survey; CI, confidence interval; SSRIs, selective serotonin reuptake inhibitor(s); PA, physical activity; OPA, occupation-related PA; TPA, transportation-related PA; LTPA, leisure-time PA.

SI conversion factor: To convert cotinine to nanomoles per liter, multiply by 5.675.

^*^ No opioid use was used as comparison.

^†^ Unadjusted.

^‡^ Adjusted for age (continuous), sex (male or female), race/ethnicity (non-Hispanic White, non-Hispanic Black, Mexican American, or other races).

^§^ Adjusted for covariates in the model 2 plus education (less than high school, high school, or more than high school), poverty-income ratio (≤130%,130%-300%, or >300%), any insurance (no or yes), alcohol user (never, former, mild, moderate, or heavy), cotinine (continuous), and survey cycle (2007-2008 to 2019-2020).

**Table S10. Associations between PA and long-term use of prescription opioids among adults without taking benzodiazepines or SSRIs** **antidepressants in NHANES, 2007 to 2020^*^**

|  | **Odds ratio (95% CI)** | | | | | |
| --- | --- | --- | --- | --- | --- | --- |
|  | **Weaker than morphine** | | | **Equivalent to morphine or stronger** | | |
|  | **Model 1^†^** | **Model 2^‡^** | **Model 3^§^** | **Model 1^†^** | **Model 2^‡^** | **Model 3^§^** |
| **Total PA** |  |  |  |  |  |  |
| Insufficient | 1.00 (Ref) | 1.00 (Ref) | 1.00 (Ref) | 1.00 (Ref) | 1.00 (Ref) | 1.00 (Ref) |
| Sufficient | **0.42 (0.29 – 0.61)** | **0.56 (0.38 – 0.88)** | **0.59 (0.40 – 0.87)** | **0.50 (0.40 – 0.61)** | **0.59 (0.48 – 0.73)** | **0.65 (0.53 – 0.81)** |
| **OPA** |  |  |  |  |  |  |
| Insufficient | 1.00 (Ref) | 1.00 (Ref) | 1.00 (Ref) | 1.00 (Ref) | 1.00 (Ref) | 1.00 (Ref) |
| Sufficient | 0.90 (0.61 – 1.31) | 1.11 (0.76 – 1.63) | 1.13 (0.78 – 1.65) | **0.80 (0.65 – 0.98)** | 0.91 (0.73 – 1.13) | 0.90 (0.73 – 1.11) |
| **TPA** |  |  |  |  |  |  |
| Insufficient | 1.00 (Ref) | 1.00 (Ref) | 1.00 (Ref) | 1.00 (Ref) | 1.00 (Ref) | 1.00 (Ref) |
| Sufficient | **0.40 (0.24 – 0.68)** | **0.48 (0.28 – 0.81)** | **0.48 (0.29 – 0.81)** | 0.88 (0.66 – 1.16) | 1.06 (0.80 – 1.40) | 0.96 (0.72 – 1.28) |
| **LTPA** |  |  |  |  |  |  |
| Insufficient | 1.00 (Ref) | 1.00 (Ref) | 1.00 (Ref) | 1.00 (Ref) | 1.00 (Ref) | 1.00 (Ref) |
| Sufficient | **0.38 (0.23 – 0.62)** | **0.48 (0.29 – 0.77)** | **0.50 (0.32 – 0.80)** | **0.42 (0.31 – 0.57)** | **0.48 (0.36 – 0.64)** | **0.57 (0.42 – 0.77)** |

Abbreviations: NHANES, National Health and Nutrition Examination Survey; CI, confidence interval; SSRIs, selective serotonin reuptake inhibitor(s); PA, physical activity; OPA, occupation-related PA; TPA, transportation-related PA; LTPA, leisure-time PA.

SI conversion factor: To convert cotinine to nanomoles per liter, multiply by 5.675.

^*^ No opioid use was used as comparison.

^†^ Unadjusted.

^‡^ Adjusted for age (continuous), sex (male or female), race/ethnicity (non-Hispanic White, non-Hispanic Black, Mexican American, or other races).

^§^ Adjusted for covariates in the model 2 plus education (less than high school, high school, or more than high school), poverty-income ratio (≤130%,130%-300%, or >300%), any insurance (no or yes), alcohol user (never, former, mild, moderate, or heavy), cotinine (continuous), and survey cycle (2007-2008 to 2019-2020).

**Table S11. Associations between PA and short-term use of prescription opioids among adults in NHANES, 2007 to 2020^*^**

|  | **Odds ratio (95% CI)** | | |
| --- | --- | --- | --- |
|  | **Model 1^†^** | **Model 2^‡^** | **Model 3^§^** |
| **Total PA** |  |  |  |
| Insufficient | 1.00 (Ref) | 1.00 (Ref) | 1.00 (Ref) |
| Sufficient | **1.97 (1.31 – 2.95)** | **1.71 (1.12 – 2.63)** | **1.74 (1.14 – 2.65)** |
| **OPA** |  |  |  |
| Insufficient | 1.00 (Ref) | 1.00 (Ref) | 1.00 (Ref) |
| Sufficient | 1.27 (0.83 – 1.94) | 1.15 (0.74 – 1.78) | 1.11 (0.71 – 1.74) |
| **TPA** |  |  |  |
| Insufficient | 1.00 (Ref) | 1.00 (Ref) | 1.00 (Ref) |
| Sufficient | 0.89 (0.51 – 1.53) | 0.82 (0.47 – 1.42) | 0.78 (0.44 – 1.37) |
| **LTPA** |  |  |  |
| Insufficient | 1.00 (Ref) | 1.00 (Ref) | 1.00 (Ref) |
| Sufficient | 0.98 (0.65 – 1.46) | 0.86 (0.56 – 1.31) | 0.89 (0.57 – 1.40) |

Abbreviations: NHANES, National Health and Nutrition Examination Survey; CI, confidence interval; PA, physical activity; OPA, occupation-related PA; TPA, transportation-related PA; LTPA, leisure-time PA.

SI conversion factor: To convert cotinine to nanomoles per liter, multiply by 5.675.

^*^ Defined prescription opioid use for <30 days as short-term use, and no opioid use was used as comparison.

^†^ Unadjusted.

^‡^ Adjusted for age (continuous), sex (male or female), race/ethnicity (non-Hispanic White, non-Hispanic Black, Mexican American, or other races).

^§^ Adjusted for covariates in the model 2 plus education (less than high school, high school, or more than high school), poverty-income ratio (≤130%,130%-300%, or >300%), any insurance (no or yes), alcohol user (never, former, mild, moderate, or heavy), cotinine (continuous), and survey cycle (2007-2008 to 2019-2020).

**Table S12. Associations between PA and long-term use of prescription opioids among adults in NHANES, 2007 to 2020^*^**

|  | **Odds ratio (95% CI)** | | | | | |
| --- | --- | --- | --- | --- | --- | --- |
|  | **Weaker than morphine** | | | **Equivalent to morphine or stronger** | | |
|  | **Model 1^†^** | **Model 2^‡^** | **Model 3^§^** | **Model 1^†^** | **Model 2^‡^** | **Model 3^§^** |
| **Total PA** |  |  |  |  |  |  |
| Insufficient | 1.00 (Ref) | 1.00 (Ref) | 1.00 (Ref) | 1.00 (Ref) | 1.00 (Ref) | 1.00 (Ref) |
| Sufficient | **0.43 (0.31 – 0.58)** | **0.57 (0.42 – 0.79)** | **0.62 (0.45 – 0.85)** | **0.46 (0.39 – 0.56)** | **0.53 (0.44 – 0.65)** | **0.60 (0.49 – 0.72)** |
| **OPA** |  |  |  |  |  |  |
| Insufficient | 1.00 (Ref) | 1.00 (Ref) | 1.00 (Ref) | 1.00 (Ref) | 1.00 (Ref) | 1.00 (Ref) |
| Sufficient | 0.86 (0.62 – 1.20) | 1.08 (0.77 – 1.52) | 1.10 (0.79 – 1.54) | **0.75 (0.63 – 0.90)** | 0.84 (0.69 – 1.01) | 0.84 (0.70 – 1.01) |
| **TPA** |  |  |  |  |  |  |
| Insufficient | 1.00 (Ref) | 1.00 (Ref) | 1.00 (Ref) | 1.00 (Ref) | 1.00 (Ref) | 1.00 (Ref) |
| Sufficient | **0.45 (0.28 – 0.72)** | **0.54 (0.34 – 0.86)** | **0.53 (0.34 – 0.84)** | **0.75 (0.57 – 0.98)** | 0.88 (0.67 – 1.16) | 0.81 (0.61 – 1.08) |
| **LTPA** |  |  |  |  |  |  |
| Insufficient | 1.00 (Ref) | 1.00 (Ref) | 1.00 (Ref) | 1.00 (Ref) | 1.00 (Ref) | 1.00 (Ref) |
| Sufficient | **0.35 (0.23 – 0.54)** | **0.44 (0.29 – 0.68)** | **0.49 (0.32 – 0.73)** | **0.42 (0.33 – 0.54)** | **0.47 (0.37 – 0.60)** | **0.57 (0.44 – 0.73)** |

Abbreviations: NHANES, National Health and Nutrition Examination Survey; CI, confidence interval; PA, physical activity; OPA, occupation-related PA; TPA, transportation-related PA; LTPA, leisure-time PA.

SI conversion factor: To convert cotinine to nanomoles per liter, multiply by 5.675.

^*^ Defined prescription opioid use for ≥30 days as long-term use, and no opioid use was used as comparison.

^†^ Unadjusted.

^‡^ Adjusted for age (continuous), sex (male or female), race/ethnicity (non-Hispanic White, non-Hispanic Black, Mexican American, or other races).

^§^ Adjusted for covariates in the model 2 plus education (less than high school, high school, or more than high school), poverty-income ratio (≤130%,130%-300%, or >300%), any insurance (no or yes), alcohol user (never, former, mild, moderate, or heavy), cotinine (continuous), and survey cycle (2007-2008 to 2019-2020).

**Table S13. E-values for the associations between PA and patterns of prescription opioid use**

|  | **OR (95% CI)** | **E-value^*^** |
| --- | --- | --- |
| **Opioid use** |  |  |
| Total PA | 0.68 (0.56 – 0.81) | 2.30 (1.78) |
| OPA | 0.93 (0.79 – 1.10) | 1.36 (1.00) |
| TPA | 0.73 (0.58 – 0.92) | 2.08 (1.39) |
| LTPA | 0.60 (0.48 – 0.75) | 2.72 (2.00) |
| **Short-term use of opioids** |  |  |
| Total PA | 1.64 (1.11 – 2.42) | 2.66 (1.46) |
| OPA | 1.07 (0.71 – 1.59) | 1.34 (1.00) |
| TPA | 0.68 (0.41 – 1.15) | 2.30 (1.00) |
| LTPA | 1.02 (0.67 – 1.57) | 1.16 (1.00) |
| **Long-term use of opioids weaker than morphine** |  |  |
| Total PA | 0.61 (0.44 – 0.85) | 2.66 (1.63) |
| OPA | 1.11 (0.79 – 1.55) | 1.46 (1.00) |
| TPA | 0.55 (0.35 – 0.86) | 3.04 (1.60) |
| LTPA | 0.47 (0.31 – 0.72) | 3.68 (2.12) |
| **Long-term use of opioids equivalent to morphine or stronger** |  |  |
| Total PA | 0.57 (0.47 – 0.70) | 2.90 (2.21) |
| OPA | 0.83 (0.68 – 1.01) | 1.70 (1.00) |
| TPA | 0.84 (0.63 – 1.12) | 1.67 (1.00) |
| LTPA | 0.52 (0.41 – 0.68) | 3.26 (2.30) |

^*^ Data presented as E-values for point estimate and for confidence interval closest to null.

Abbreviations: OR, odds ratio; CI, confidence interval; PA, physical activity; OPA, occupation-related PA; TPA, transportation-related PA; LTPA, leisure-time PA.

**Table S14. Missing variables and multiple imputation methods**

| **Variables (raw)** | **Missing（%）** | **Imputation methods** |
| --- | --- | --- |
| Age (years) | 0 | - |
| Gender | 0 | - |
| Race/Ethnicity | 0 | - |
| Family PIR | 4206 (10.72%) | Predictive mean matching |
| Education level | 465 (1.19%) | Proportional odds model |
| Cotinine (ng/mL) | 2366 (6.03%) | Predictive mean matching |
| Alcohol user^*^ | 6319 (16.11%) | Proportional odds model |
| Insurance | 66 (0.17%) | Logistic regression |
| Average missing (%): 6.84% | | |

^*^ Created using alcohol questionnaire (including never, former, mild, moderate, or heavy; see main text). Family PIR (continuous), Education level (less than high school, high school, or more than high school), Cotinine (continuous).

**Table S15. Associations between PA and prescription opioid use by multiple imputation among adults in NHANES, 2007 to 2020**

|  | **Odds ratio (95% CI)** | | |
| --- | --- | --- | --- |
|  | **Model 1*** | **Model 2^†^** | **Model 3^‡^** |
| **Total PA** |  |  |  |
| Insufficient | 1.00 (Ref) | 1.00 (Ref) | 1.00 (Ref) |
| Sufficient | **0.55 (0.47 – 0.64)** | **0.63 (0.54 – 0.74)** | **0.68 (0.59 – 0.79)** |
| **OPA** |  |  |  |
| Insufficient | 1.00 (Ref) | 1.00 (Ref) | 1.00 (Ref) |
| Sufficient | **0.86 (0.74 – 0.99)** | 0.95 (0.82 – 1.10) | 0.95 (0.83 – 1.08) |
| **TPA** |  |  |  |
| Insufficient | 1.00 (Ref) | 1.00 (Ref) | 1.00 (Ref) |
| Sufficient | **0.67 (0.56 – 0.79)** | **0.77 (0.65 – 0.92)** | **0.73 (0.61 – 0.87)** |
| **LTPA** |  |  |  |
| Insufficient | 1.00 (Ref) | 1.00 (Ref) | 1.00 (Ref) |
| Sufficient | **0.47 (0.39 – 0.58)** | **0.53 (0.44 – 0.65)** | **0.60 (0.49 – 0.74)** |

Abbreviations: NHANES, National Health and Nutrition Examination Survey; CI, confidence interval; PA, physical activity; OPA, occupation-related PA; TPA, transportation-related PA; LTPA, leisure-time PA.

SI conversion factor: To convert cotinine to nanomoles per liter, multiply by 5.675.

^*^ Unadjusted.

^†^ Adjusted for age (continuous), sex (male or female), race/ethnicity (non-Hispanic White, non-Hispanic Black, Mexican American, or other races).

^‡^ Adjusted for covariates in the model 2 plus education (less than high school, high school, or more than high school), poverty-income ratio (≤130%,130%-300%, or >300%), any insurance (no or yes), alcohol user (never, former, mild, moderate, or heavy), cotinine (continuous), and survey cycle (2007-2008 to 2019-2020).

**Table S16. Associations between PA and short-term use of prescription opioids by multiple imputation among adults in NHANES, 2007 to 2020^*^**

|  | **Odds ratio (95% CI)** | | |
| --- | --- | --- | --- |
|  | **Model 1^†^** | **Model 2^‡^** | **Model 3^§^** |
| **Total PA** |  |  |  |
| Insufficient | 1.00 (Ref) | 1.00 (Ref) | 1.00 (Ref) |
| Sufficient | **1.84 (1.37 – 2.49)** | **1.56 (1.14 – 2.15)** | **1.58 (1.15 – 2.17)** |
| **OPA** |  |  |  |
| Insufficient | 1.00 (Ref) | 1.00 (Ref) | 1.00 (Ref) |
| Sufficient | 1.28 (0.95 – 1.72) | 1.15 (0.85 – 1.57) | 1.11 (0.80 – 1.52) |
| **TPA** |  |  |  |
| Insufficient | 1.00 (Ref) | 1.00 (Ref) | 1.00 (Ref) |
| Sufficient | 0.67 (0.43 – 1.07) | 0.61 (0.39 – 0.98) | **0.57 (0.35 – 0.92)** |
| **LTPA** |  |  |  |
| Insufficient | 1.00 (Ref) | 1.00 (Ref) | 1.00 (Ref) |
| Sufficient | 1.17 (0.84 – 1.61) | 1.00 (0.71 – 1.39) | 1.05 (0.72 – 1.51) |

Abbreviations: NHANES, National Health and Nutrition Examination Survey; CI, confidence interval; PA, physical activity; OPA, occupation-related PA; TPA, transportation-related PA; LTPA, leisure-time PA.

SI conversion factor: To convert cotinine to nanomoles per liter, multiply by 5.675.

^*^ No opioid use was used as comparison.

^†^ Unadjusted.

^‡^ Adjusted for age (continuous), sex (male or female), race/ethnicity (non-Hispanic White, non-Hispanic Black, Mexican American, or other races).

^§^ Adjusted for covariates in the model 2 plus education (less than high school, high school, or more than high school), poverty-income ratio (≤130%,130%-300%, or >300%), any insurance (no or yes), alcohol user (never, former, mild, moderate, or heavy), cotinine (continuous), and survey cycle (2007-2008 to 2019-2020).

**Table S17. Associations between PA and long-term use of prescription opioids by multiple imputation among adults in NHANES, 2007 to 2020^*^**

|  | **Odds ratio (95% CI)** | | | | | |
| --- | --- | --- | --- | --- | --- | --- |
|  | **Weaker than morphine** | | | **Equivalent to morphine or stronger** | | |
|  | **Model 1^†^** | **Model 2^‡^** | **Model 3^§^** | **Model 1^†^** | **Model 2^‡^** | **Model 3^§^** |
| **Total PA** |  |  |  |  |  |  |
| Insufficient | 1.00 (Ref) | 1.00 (Ref) | 1.00 (Ref) | 1.00 (Ref) | 1.00 (Ref) | 1.00 (Ref) |
| Sufficient | **0.43 (0.34 – 0.54)** | **0.57 (0.45 – 0.73)** | **0.61 (0.48 – 0.78)** | **0.46 (0.38 – 0.54)** | **0.54 (0.45 – 0.64)** | **0.59 (0.50 – 0.70)** |
| **OPA** |  |  |  |  |  |  |
| Insufficient | 1.00 (Ref) | 1.00 (Ref) | 1.00 (Ref) | 1.00 (Ref) | 1.00 (Ref) | 1.00 (Ref) |
| Sufficient | 0.83 (0.63 – 1.09) | 1.04 (0.79 – 1.38) | 1.05 (0.79 – 1.39) | **0.77 (0.65 – 0.92)** | 0.87 (0.72 – 1.04) | 0.87 (0.73 – 1.03) |
| **TPA** |  |  |  |  |  |  |
| Insufficient | 1.00 (Ref) | 1.00 (Ref) | 1.00 (Ref) | 1.00 (Ref) | 1.00 (Ref) | 1.00 (Ref) |
| Sufficient | **0.51 (0.36 – 0.72)** | **0.62 (0.43 – 0.89)** | **0.61 (0.43 – 0.87)** | **0.74 (0.59 – 0.91)** | 0.89 (0.72 – 1.11) | 0.83 (0.66 – 1.04) |
| **LTPA** |  |  |  |  |  |  |
| Insufficient | 1.00 (Ref) | 1.00 (Ref) | 1.00 (Ref) | 1.00 (Ref) | 1.00 (Ref) | 1.00 (Ref) |
| Sufficient | **0.32 (0.21 – 0.47)** | **0.40 (0.27 – 0.59)** | **0.43 (0.29 – 0.62)** | **0.41 (0.32 – 0.51)** | **0.47 (0.37 – 0.58)** | **0.55 (0.44 – 0.69)** |

Abbreviations: NHANES, National Health and Nutrition Examination Survey; CI, confidence interval; PA, physical activity; OPA, occupation-related PA; TPA, transportation-related PA; LTPA, leisure-time PA.

SI conversion factor: To convert cotinine to nanomoles per liter, multiply by 5.675.

^*^ No opioid use was used as comparison.

^†^ Unadjusted.

^‡^ Adjusted for age (continuous), sex (male or female), race/ethnicity (non-Hispanic White, non-Hispanic Black, Mexican American, or other races).

^§^ Adjusted for covariates in the model 2 plus education (less than high school, high school, or more than high school), poverty-income ratio (≤130%,130%-300%, or >300%), any insurance (no or yes), alcohol user (never, former, mild, moderate, or heavy), cotinine (continuous), and survey cycle (2007-2008 to 2019-2020).
